# Optogenetic Inhibition of Rat Anterior Cingulate Cortex Impairs the Ability to Initiate and Stay on Task

**DOI:** 10.1523/JNEUROSCI.1850-23.2024

**Published:** 2024-04-03

**Authors:** Daniela Vázquez, Sean R. Maulhardt, Thomas A. Stalnaker, Alec Solway, Caroline J. Charpentier, Matthew R. Roesch

**Affiliations:** ^1^Department of Psychology, University of Maryland, College Park, Maryland 20742; ^2^Program in Neuroscience and Cognitive Science, University of Maryland, College Park, Maryland 20742; ^3^Intramural Research Program, National Institute on Drug Abuse, Baltimore, Maryland 21224

**Keywords:** anterior cingulate cortex, attention, choice, drift diffusion, optogenetic, rat, reward, task engagement

## Abstract

Our prior research has identified neural correlates of cognitive control in the anterior cingulate cortex (ACC), leading us to hypothesize that the ACC is necessary for increasing attention as rats flexibly learn new contingencies during a complex reward-guided decision-making task. Here, we tested this hypothesis by using optogenetics to transiently inhibit the ACC, while rats of either sex performed the same two-choice task. ACC inhibition had a profound impact on behavior that extended beyond deficits in attention during learning when expected outcomes were uncertain. We found that ACC inactivation slowed and reduced the number of trials rats initiated and impaired both their accuracy and their ability to complete sessions. Furthermore, drift–diffusion model analysis suggested that free-choice performance and evidence accumulation (i.e., reduced drift rates) were degraded during initial learning—leading to weaker associations that were more easily overridden in later trial blocks (i.e., stronger bias). Together, these results suggest that in addition to attention-related functions, the ACC contributes to the ability to initiate trials and generally stay on task.

## Significance Statement

Attentional deficits and the ability to stay on task are the defining hallmarks of some of the most prevalent and disruptive neuropsychiatric disorders. Here, we use an optogenetic approach and computational modeling to study how within-subject modulation of the anterior cingulate cortex (ACC) impacts the ability of rats to initiate and complete a complex reward-guided decision-making task. We found that on days in which the ACC was inhibited, the ability of rats to initiate and stay on task was impaired, as well as their task accuracy and ability to complete sessions.

## Introduction

The anterior cingulate cortex (ACC) has been implicated across a plethora of cognitive functions—including attention and error detection (Rescorla, 1973; D'Esposito et al., 1995; Carter et al., 1998; Botvinick et al., 1999; Carter et al., 1999; Barch, 2001; Braver, 2001; Paus, 2001; Botvinick et al., 2004; Holroyd et al., 2004; Kerns et al., 2004; Weissman, 2004; Yeung et al., 2004; Carter and Van Veen, 2007; Aarts et al., 2008; Totah et al., 2009; Koob and Volkow, 2010; Hayden et al., 2011; Lovstad et al., 2012; Hyman et al., 2013; Narayanan et al., 2013; Laubach et al., 2015; Newman et al., 2015; Vassena et al., 2017; Wu et al., 2017; Soltani and Izquierdo, 2019; Stolyarova et al., 2019; Brockett et al., 2020; Schneider et al., 2020; Vázquez et al., 2022; Ye et al., 2023). Consistent with this work, single-neuron studies have described correlates related to these functions (Davis et al., 2000; Bryden et al., 2019; Seamans, 2021). Our own work has shown that firing in the ACC correlates with changes in attention proposed by the Pearce and Hall model of associative learning (Bryden et al., 2011; Roesch et al., 2012; Vázquez et al., 2020). In this model, the attention given to a cue on subsequent trials is a product of the average unsigned prediction errors generated over trials. Unsigned prediction errors reflect the degree to which an outcome is unexpected and results from the difference between the value of expected reward and the actual outcome (Pearce and Hall, 1980). We found that ACC firing at the start of trials—from trial onset to completion of the behavioral response—correlated with changes in attention during flexible learning of response–outcome contingencies on trials following reward prediction errors (Bryden et al., 2011). In another study, we found that the disruption of ACC signaling after cocaine self-administration correlated with decision-making impairments that were tightly linked to attentional deficits (Vázquez et al., 2020).

These studies suggest that the ACC is critical for cognitive functions necessary for task performance. To test this hypothesis, we used optogenetics to transiently inactivate the ACC from the start of the trial until the end of the behavioral response. During the performance of this task, the reward value was manipulated by independently varying the delay to and size of reward. Optimal task performance required rats to detect unexpected changes in reward and update behavior accordingly to select the more favorable reward outcome on free-choice trials while maintaining accuracy on previously learned stimulus–response associations (i.e., forced-choice trials).

ACC inactivation had a profound impact on the rats' basic ability to initiate and complete trials—with rats being slower to initiate trials and initiating and completing fewer trials—which resulted in reduced session completion and obtaining less reward overall. ACC inactivation also impaired free-choice performance for contingencies associated with learning in the first block of trials (i.e., original discrimination). Inactivation did not impact the overall movement time (MT) or reduce time spent consuming reward, suggesting that the observed behavioral impairments during inhibition days were not a result of motor or motivational deficits.

Finally, we fit a drift–diffusion model (DDM) to our data to better understand the possible mechanisms giving rise to altered free-choice performance. DDMs are sequential sampling models that allow us to deconstruct choice and response time behaviors in the service of understanding decision-making in terms of information accumulation, decision thresholds, and biases (Ratcliff and Rouder, 1998; Voss et al., 2004; Bogacz et al., 2006; Gold and Shadlen, 2007; Ratcliff and McKoon, 2008; Forstmann et al., 2016; Ratcliff et al., 2016; Shinn et al., 2020; Lei and Solway, 2022). This model posits that during the performance of a two-choice task, animals continually accumulate evidence for each alternative—starting from a point that could be biased toward one of the two alternatives and reaching a decision once the evidence for one of the two alternatives reaches a sufficient threshold. We found that ACC inhibition reduced the rate at which rats accumulated evidence and created stronger biases in later blocks (Lei and Solway, 2022). Together, these findings suggest that optogenetic inhibition not only impairs attention and the ability to accumulate knowledge to support optimal decision-making, but it also drastically impedes rats' ability to initiate and sustain task performance.

## Materials and Methods

### Subjects

Male Long–Evans rats (*n *= 10) were obtained at ∼2–3 months of age from Charles River Laboratories, weighing in the range of 150–200 g. Rats were tested at the University of Maryland (UMD), in accordance with UMD and NIH guidelines. During the behavioral testing, food was available *ad libitum*; water intake was restricted to ensure motivation for task performance.

### Experimental design

**Figure 1.**
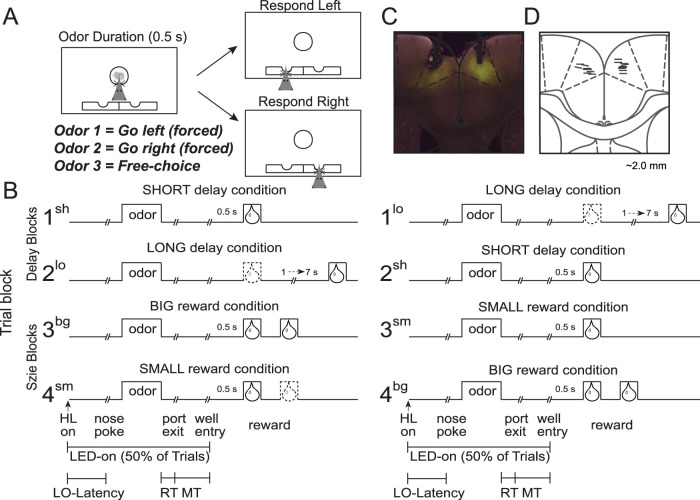
Task schematics and histology. ***A***, ***B***, Schematic of the behavioral apparatus and task—the experimental paradigm allows for independent manipulations of reward value through variations of delay (Blocks 1 and 2) and size (Blocks 3 and 4) across four 60-trial blocks. Upward deflections reflect onset of cues and delivery of reward. On each trial, rats nose-poked into a central odor port upon illumination of the houselights (HL) to receive one of the three odors and then responded in the corresponding fluid well to receive reward. One odor signaled reward in the left well (forced-choice), another indicated reward in the right well (forced-choice), and a third odor signaled reward at either well (free-choice). The time of LED illumination and behavioral measures, LO latency (houselight on to odor port entry), RT (odor offset to odor port exit), and MT (odor port exit to well entry) are depicted along the *x*-axis. ***C***, ***D***, Histological verification of virus (NpHR-eYFP). and optical fiber placements in the ACC. Horizontal lines denote the bottom and angle of the optical fibers.

Rats were trained for a month on a reward-guided decision-making task (Fig. 1; for more details, see Bryden et al., 2011; Vázquez et al., 2020). In this task, houselight illumination indicated the beginning of a new trial. On each trial, rats had to nose-poke into the odor port following houselight illumination, maintaining the nose-poke for 500 ms resulting in the delivery of a directional odor cue. One odor instructed the rat to go to the left fluid well to receive reward (forced-choice, left), a second odor instructed the rat to go to the right fluid well to receive reward (forced-choice, right), and a third odor indicated that the rat could obtain reward at either well (free-choice). On forced-choice trials, if the rat went to the incorrect well, the reward was not delivered. Odors were presented in a pseudorandom sequence and were counterbalanced across rats.

A completed session consists of four 60-trial blocks. At the start of each session, one well was randomly assigned to have a short delay to reward (0.5 s) and the other a long delay (1–7 s; Fig. 1*A,B*, Block 1). We refer to the side-value contingencies defined in the first block of trials (which are repeated in the third block of trials) as the “original” learned associations. In the second block of trials, these contingencies were interchanged (Fig. 1*B*, Block 2). As this is a reversal of the originally learned contingencies, we refer to the side-value contingencies defined in the second block of trials (which are repeated in the fourth block of trials) as the “reversal” of the originally learned associations. The length of the “long” delay condition began at a delay length of 1 s and increased by 1 s every time the rat chose the delayed side on free-choice odor trials (up to a maximum of 7 s); gradual increases better convey reward delays as opposed to outright omission or errant behavior and promote continuous task performance. The delays on forced-choice trials were yoked to the delay on free-choice trials. During the final two blocks of the task, the delay preceding the reward delivery was held constant (0.5 s on both sides) while manipulating the size of the expected reward (Fig. 1*B*, Blocks 3 and 4). Throughout the task, the reward consisted of a single 0.05 ml bolus of 10% sucrose solution; on size trials, large reward consisted of an additional bolus being delivered 0.5 s after the first bolus. As in our previous publications, reward delay was always manipulated in the first two blocks, and reward size was manipulated in the final two blocks to promote session completion (Bryden et al., 2011; Roesch et al., 2012; Vázquez et al., 2020).

Following training, all rats underwent a surgical procedure to receive virus injections and fiber-optic implants. We bilaterally injected halorhodopsin (AAV-CaMKIIa-eNPHR3.0-EYFP from the UNC Vector Core; 0.6 µl per hemisphere) and implanted optical fibers into the rat ACC (coordinates, 0.2 mm anterior to the bregma, ±0.5 mm lateral, and 1.0 mm ventral to the brain). The virus we used has been reliably established in optogenetics literature (Zhang et al., 2007; Gradinaru et al., 2008; Zhao et al., 2008; Fenno et al., 2017)—across both in vivo and in vitro experiments—as an inhibitory opsin and one that specifically inhibits pyramidal cells as a result of its CaMKII promoter (Han and Boyden, 2007; Zhang et al., 2007; Arrenberg et al., 2009; Calu et al., 2013; Stefanik et al., 2013; Chang et al., 2016; Gardner et al., 2017; Gholami and Sayyah, 2018; Hart et al., 2020). Moreover, patch-clamp experiments using the same halorhodopsin virus we used have shown that yellow light illumination results in clear inhibition of excitatory postsynaptic currents, with no rebound excitation (de Lima et al., 2022).

Following a week of recovery after surgery, rats performed the previously described decision-making task. Every 2 d, we alternated between using a blue (control days) or yellow (inhibition days) LED to optogenetically inactivate the ACC, while rats performed the aforementioned task (Fig. 1*C*,*D*). Throughout the task, LED delivery randomly occurred on 50% of trials—and lasted from the onset of houselights until completion of the behavioral response (e.g., “LED-on” period—the epoch from houselight on to well entry—Fig. 1*B*). During sessions, LED light was shielded so as not to be a distracting stimulus—electrical tape was wrapped around the sleeves connecting the optical fibers to the ferrules. Regardless, since our control was a blue LED light, if the LEDs were distracting or reinforcing in some way, then that would be controlled for as well (Wikenheiser et al., 2017); many studies, both in vivo and in vitro, which do optical excitation and inhibition in the same cells, use both halorhodopsin and channelrhodopsin; because of the difference in excitation wavelengths, these two opsins can be coexpressed (e.g., only yellow light will activate halorhodopsin—blue light will have no impact; Gradinaru et al., 2007; Han and Boyden, 2007; Zhang et al., 2007; Sharpe et al., 2017; Chang et al., 2018). Furthermore—as we will describe below—inhibition also impacted trials when the LED was not illuminated, demonstrating that behavioral changes observed during inhibition sessions cannot be a product of the physical presence of LED illumination. We used PlexBright LED Optogenetics Modules controlled by PlexBright Dual LED commutators to deliver light (blue, 465 nm; yellow, 590 nm) via Plexon's 200 µm core optical fibers.

### Behavioral analysis

The behavior for each session was analyzed by calculating the total number of reward trials, percentage of initiated trials (i.e., how often rats responded to houselights), total number of incomplete trials (i.e., rats did not maintain hold centrally or respond to one of the two wells), percentage of correct response on forced-choice trials (i.e., the number of trials the animal correctly responded to the side corresponding to the directional odor), the percentage of trials rats chose a high-valued condition (i.e., short delay, large reward) on free-choice trials, reaction times (RT; odor offset to odor port exit; Fig. 1*B*), MTs (port exit to well entry Fig. 1*B*), time spent in the fluid well after reward onset, and light-on (LO) latencies (latency to enter odor port upon illumination of houselights; Fig. 1*B*). Behavioral analyses were computed for each individual session (separated by control and inhibition days) and then averaged across sessions within each group. Multifactor analysis of variance (ANOVA) statistics with relevant factors were conducted on the abovementioned behavioral measures. Factors included experimental manipulation (inhibition vs control session), LED (on vs off), phase (early vs late in learning), block type (delay vs size), discrimination (original vs learning), and value (high vs low). Post hoc *t* tests corrected for multiple comparisons were used to explore significant interaction terms in the ANOVAs.

### DDM

We fit the DDM (https://osf.io/dwm8p/) using a multilevel Bayesian structure in the Stan programming language (Stan Development Team) using accuracy and RT on free-choice trials after learning (excludes the first 20 trials of each block) to better characterize choice selection differences that emerged as a result of inhibition in terms of evidence accumulation, decision threshold and starting bias (Ratcliff and Rouder, 1998; Voss et al., 2004; Bogacz et al., 2006; Gold and Shadlen, 2007; Ratcliff and McKoon, 2008; Forstmann et al., 2016; Ratcliff et al., 2016; Shinn et al., 2020; Lei and Solway, 2022). This allowed us to partition variance along the experimental blocks that carried reward size and delay contingencies and group-level and session effects. We used Stan because of its efficient Hamiltonian Monte Carlo/no-U-turn Markov chain Monte Carlo (MCMC) sampler (Hoffman and Gelman, 2014), especially in terms of posterior effective sample size, as well as its ability to fit group data using multilevel models, which are known to provide more reliable parameters estimates through partial pooling. Stan allows us to both fit multilevel models and perform inference efficiently. We ran four MCMC chains with a 1,000 iteration warm-up and 3,000 additional iterations for estimating the posterior. The *R*^^^ statistic was <1.02 for all parameters, and trace plots were desirable, indicating good convergence between the chains. Group- and individual-level parameters were created per block type (delay or size) and condition (control or inhibition). The comparison of group-level parameter distributions sheds light on the variance partitioning between block and condition. Each parameter set (*θ*) contains four components of the DDM—boundary separation (*α*), nondecision time (*τ*), starting bias (*β*), and drift rate (*δ*). Boundary separation gives width to the criterion, representing the threshold of evidence required to make a decision. Nondecision time is the fixed processing time before and after decision-related processes. Starting bias moves the initial starting point toward the criterion, and drift rate is the rate of evidence accumulation and represents signal strength toward the criterion. We used uninformative group-level Gaussian priors *N*(0, 20) for the mean and standard deviation of boundary separation, drift rate, and starting bias and *N*(0, 1) for the nondecision time.

The power of this DDM analysis is that it separates the different components of the decision process that impact raw choices and RTs. The model parameters are constrained in a nonlinear manner by the behavioral data, and importantly, differences in raw choices and RTs may be nonsignificant between groups, while at the same time differences in model parameters may be significant—one of the primary goals in fitting the model is to increase the statistical power for making inferences about specific dimensions of decision-making (captured by the parameters), which are conflated when looking at raw behavioral data. This has previously been demonstrated through simulation (White et al., 2010), and it has been shown that mapping between patterns of behavioral (choice and RT) data and model parameters is not always intuitive, and the latter cannot be inferred from the former without fitting the model (Lerche and Voss, 2020).

## Results

### Inhibition of the ACC reduced the number of trials and sessions completed

**Figure 2.**
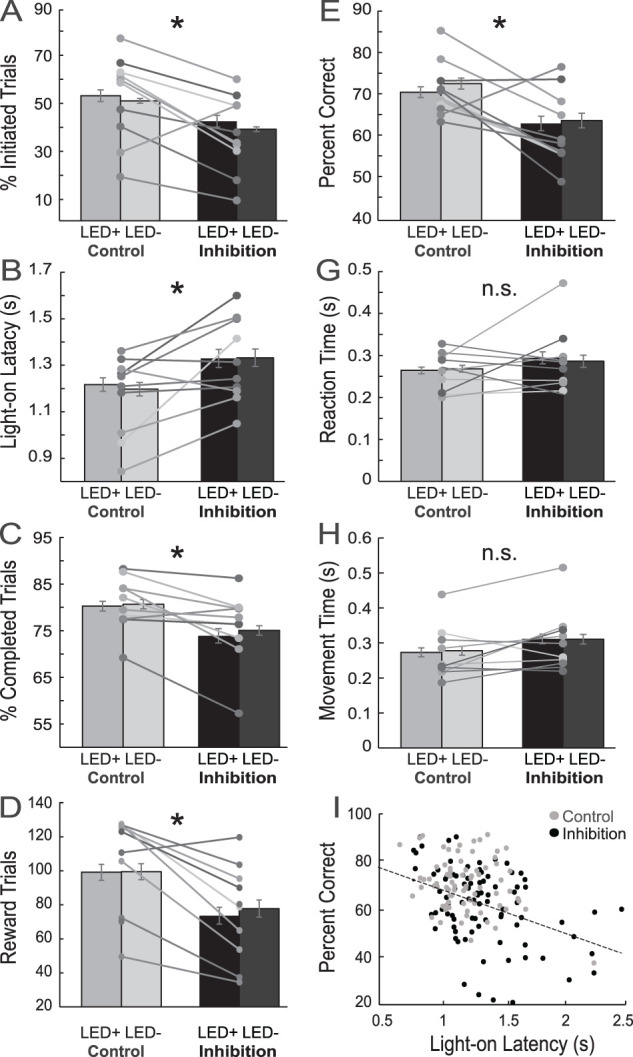
Behavioral analyses across all sessions, comparing inhibition to control days. Bar graphs are further broken into LED-on (labeled “LED+”) versus LED-off (“LED-”) trials. ***A***, Percentage of times that rats initiated trials. ***B***, Average LO latency denotes the latency to enter odor port upon houselight illumination (which indicates the beginning of a new trial). ***C***, The average percentage of trials rats completed each session, where we define completed trials as trials in which rats executed the entire behavioral sequence to completion—maintaining their nose-poke in the central odor port for one second and then responding to one of the two fluid wells (regardless of whether correctly or incorrectly) within the allotted 3 s for trial completion. ***D***, Average number of rewarded trials. ***E***, Percentage of correct forced-choice trials. ***F***, Percentage of times rats chose high-value reward on free-choice trials. ***F***, Average RTs (odor offset to odor port exit). ***G***, Average MTs (port exit to well entry). ***H***, Correlation between the percentage of correct response and LO latencies on inhibition (black) and control (gray) days. For all graphs, the lines indicate the averages of individual rats across each condition (control and inhibition, *n* = 10); bar graphs represent the average across all sessions within each condition. Asterisks indicate significance (*p* < 0.05); ANOVAs and post hoc *t* tests (*p* < 0.05) were used to determine differences between inhibition and control day (see Results).

Rats performed the task for 92 inhibition sessions and 89 control sessions. As previously mentioned, LED illumination occurred on 50% of trials, lasting from houselight on to well entry (Fig. 1*B*)—as this is the epoch during which we saw firing related to the initiation of trials and attention during recordings in our previous studies (Bryden et al., 2011; Vázquez et al., 2020). Furthermore, it is during that epoch that we have observed changes in firing due to chronic cocaine self-administration (Vázquez et al., 2020). With Figure 2, and the section below, we will demonstrate that ACC inhibition had a profound impact on the rats' basic ability to initiate and complete trials. We will first show, by conducting our initial analyses across all behavioral sessions—including those that were incomplete (i.e., sessions in which rats did not complete all four blocks in the allotted 2 h per session)—that general measures of task performance common across blocks (i.e., the percentage of initiated and completed trials, latencies to initiate trials, number of rewarded trials per session, and forced-choice accuracy) were impaired not only on LED-on trials but also LED-off trials. That is, when we included LED (on vs off) as one of the factors in the ANOVA, we found no significant main or interaction effects with the LED being on versus off. For the percentage of initiated trials, LO latencies, the percentage of completed trials, and number of rewards obtained, we found the main effects of session-type (inhibition vs control sessions; *F*_(1,339)_ > 14.4; *p* < 0.0002) but no significant main or interaction effects (*F*_(1,339)_ > 1.52; *p* > 0.22) with LEDs being on versus off. To summarize these findings, we impaired the rats on both LED-on and LED-off trials during inhibition sessions compared with that during the control sessions. Finding no main or interaction effects with LED on versus off across measures, we collapsed the data across these trial types in the analysis below.

Figure 2*A* illustrates the percentage of trials that rats initiated, averaged across sessions (bars) and for each individual rat (dots) during inhibition (right) and control (left) sessions. An initiated trial is defined by nose-poking into the odor port upon illumination of the houselights (Fig. 1*B*; houselight on). If the rat did not initiate a trial within 5 s, then the houselights turned off for a 2 s time interval. On average, during inhibition of the ACC, there were significantly fewer trials initiated in response to the houselights (Fig. 2*A*; *t*_(173)_ = 3.63; *p* = 0.0004).

Not only did rats initiate trials less frequently, but they were also slower to respond to the houselights when they did initiate trials (i.e., nose-poking upon houselight illumination). Figure 2*B* illustrates the latency at which rats responded to the houselights on initiated trials (i.e., epoch between houselight onset and rat entry into the central odor port within the 5 s of houselight illumination; Fig. 1*B*, LO latency) during inhibition (right) and control (left) sessions. In our previous research, we have found that this measure serves as a proxy for how strongly attentional processes are being engaged (Bryden et al., 2011; Roesch et al., 2012; Vázquez et al., 2020). On average, during inhibition sessions, latencies were significantly longer, indicating that rats were slower to initiate trials when the ACC was inhibited (*t*_(173)_ = 2.80; *p* = 0.006).

So far, we have shown that rats are slow to initiate trials and did so less frequently on inhibition days; additionally, ACC inhibition significantly diminished trial completion. Figure 2*C* plots the percentage of completed trials—once initiated—during inhibition (right) and control (left) sessions. Here, we define completed trials as trials in which rats executed the entire behavioral sequence to completion—maintaining their nose-poke in the central odor port for 1 s and then responding to one of the two fluid wells (regardless of whether correctly or incorrectly) within the allotted 3 s for trial completion (Fig. 1*B*, nose-poke to well entry). During inhibition sessions, there were significantly more failures to complete trials (*t*_(173)_ = 2.87; *p* = 0.005). As a result, during inhibition sessions, rats received fewer rewards (Fig. 2*D*; *t*_(168)_ = 3.46; *p* < 0.0001) and finished fewer sessions [inhibition, 36 (45%); control, 58 (65%); *χ*^2^* *= 7.10; *p* < 0.01].

Additionally, ACC inhibition had a deleterious impact on forced-choice trial accuracy. Recall that during forced-choice trials, rats must adhere to the learned stimulus–response contingency regardless of the reward value that it produced. Figure 2*E* plots the average percentage of the correct response over all forced-choice trials during inhibition and control sessions. On inhibition days, rats performed significantly worse (Fig. 2*E*; *t*_(168)_ = 4.23; *p* < 0.0001).

To ensure that the observed effects of ACC inhibition reducing task involvement and impacting performance was not due to a generalized impact on motivation to perform the task or motor control, we analyzed the time it took the rat to move across the different trial epochs during inhibition and control sessions averaged over trial types. RT is a measure of the amount of time rats took to exit the odor port upon odor offset (Fig. 1*B*), and MT is their measured time from odor port exit to well entry (Fig. 1*B*). Their RTs and MTs on inhibition days were not significantly different from their RTs and MTs on control days (Fig. 2*F*,*G*; RT, *t*_(168)_ = 1.31; *p* = 0.19; MT, *t*_(168)_ = 0.95; *p* = 0.34), demonstrating that ACC inhibition was not impacting motivation to perform the task (e.g., their latency to approach the well for reward was indistinguishable on control vs inhibition days) or motor control itself (e.g., their MTs were not impacted by inhibition).

Lastly, our previous study found that the behavioral measures of attention (the aforementioned “LO latencies”) were associated with better forced-choice performance across rats (Vázquez et al., 2020). Thus, here too we assessed whether there was a correlation between LO latencies and forced-choice performance; we found that faster latencies were associated with better forced-choice performance, with no differences between manipulation (Fig. 2*H*; control, *p* < 0.01; *r* = −0.31; inhibition, *p* < 0.0001; *r* = −0.41; Fisher’s *r* to *z* transformation, *z* = 0.74; *p* = 0.46)—replicating our previous findings that heightened attention is conducive to better performance.

In summary, ACC inhibition diminished and slowed the ability of rats to initiate and complete trials; it also impaired their accuracy on the task, as evinced by diminished forced-choice accuracy during inhibition sessions. Importantly, RTs and MTs averaged over all trial types and choices were unaffected by ACC inhibition, suggesting that the changes in behavior are not related to impaired motor control or decreased motivation.

### ACC inhibition disrupted free-choice selection

Following these findings, we sought to break down behavior by learning phase and reward value manipulations. To do so, we then proceeded to analyze completed sessions only (i.e., sessions in which all four blocks were completed; inhibition, 36; control, 58).

During the performance of the reward-guided decision-making task described above (Fig. 1*A*,B), we independently manipulate the reward value across two dimensions; during the first two blocks, rats choose between an immediate and delayed reward, and during the last two blocks, rats choose between a large and small reward. During the first block of trials, optimal responding requires that rats track which response direction yields the short delay and select that direction on free-choice trials (i.e., original association). After 60 trials, the location of the short delay switches to the opposite side. Thus, in order to respond optimally, rats must detect errors in reward prediction and update their free-choice behavior accordingly (i.e., reversal of originally learned contingencies). During the third block of trials, the direction that produced the long delay now produces a large reward, while the reward on the other side remains the same size. As a result, rats must switch their selection bias back in the original direction in order to obtain high-value reward more often. Finally, in the fourth block, side-value contingencies switch for the last time.

**Figure 3.**
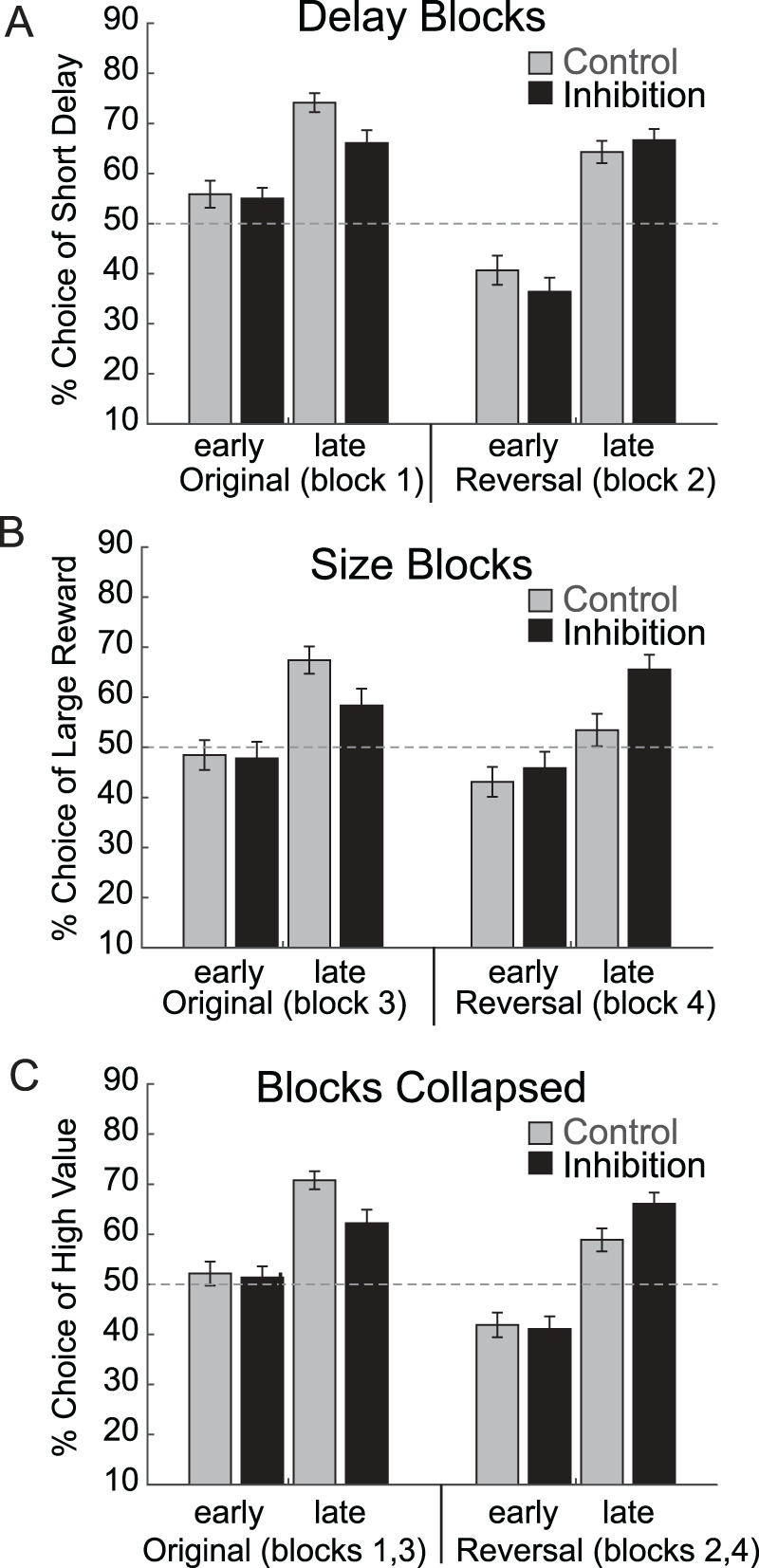
Breakdown of free-choice behavior on completed trials. ***A***, Percentage of times rats chose short-delay reward on free-choice trials during completed sessions, broken down by the learning phase (the first 10 trials in a block, e.g., “early,” versus the last 10 trials in a block, e.g., “late,” in the block once contingencies have been learned) and side-value contingencies. We refer to the side-value contingencies defined in the first block of trials (which are repeated in the third block of trials) as the “original” learned associations (e.g., Blocks 1 and 3). In the second block of trials, these contingencies were interchanged (Block 2). As this is a reversal of the originally learned contingencies, we refer to the side-value contingencies defined in the second block of trials (which are repeated in the fourth block of trials) as the “reversal” of the originally learned associations (e.g., Blocks 2 and 4). ***B***, Percentage of times rats chose large reward on free-choice trials during completed sessions, broken down by the learning phase (early in session versus late in session once contingencies have been learned) and contingencies (originally learned contingencies vs contingency reversal). ***C***, Percentage of times rats chose high-value reward (i.e., collapsing ***A***, ***B***) during completed sessions, broken down by the learning phase (early in session vs late in session once contingencies have been learned) and contingencies (originally learned contingencies vs contingency reversal).

We have previously shown that by the end of each trial block, rats learn to bias their behavior on free-choice trials toward high-value rewards and perform worse during reversals—reflecting the difficulty of overriding the originally learned associations (Bryden et al., 2011; Vazquez et al., 2020). Here too, we see that during both inhibition and control sessions, rats selected high-value rewards more often during the last 10 free-choice trials within a block (i.e., late) compared with that during the first 10 free-choice trials in the block (i.e., early). We found a main effect of phase (*F*_(1,736)_ = 73.2; *p* < 0.0001) in a four-factor ANOVA with manipulation (control vs inhibition), block type (delay vs size), reversal (original vs reversal), and phase (early vs late in learning) as factors. This is illustrated in Figure 3, which plots the percentage of times rats chose high-value reward on free-choice trials across each of the four trial blocks in the order that they were run (i.e., Blocks 1–4). Interestingly, during blocks of trials where delay was manipulated (delay blocks; Fig. 3*A*), rats chose short-delay reward less on inhibition days (black bars) compared with that on control days by the end of Block 1 (i.e., “original”) but not Block 2 (i.e., “reversal”). We observed similar effects during blocks of trials where the size was manipulated (“original” size blocks; Fig. 3*B*); however, when the size contingencies reversed, the selection of the large reward was higher during inhibition. Thus, as expected, during control sessions, discrimination performance was better than reversals; however, during reversals, the opposite tended to be true. These results are supported by a significant interaction between manipulation, phase, and reversal in the ANOVA (*F*_(1,736)_ = 4.56; *p* = 0.0331) but no significant main effect (*F*_(1,736)_ = 2.50; *p* = 0.1144) or interaction effects with the block type (*F*_(1,736)_'s < 1.59; *p*'s > 0.2095).

Since there were no significant effects of the block type, we then collapsed data across delay and size blocks (Fig. 3*C*) to further illustrate that during inhibition days, rats selected high-value reward significantly less by the end of trial blocks during the original discrimination (*t* test; *t*_(186)_ = 2.2346; *p* = 0.0266). Although the difference between groups was flipped during reversal, the comparison of control versus inhibition session only approached a significance (*t* test; *t*_(186)_ = 1.87; *p* = 0.0716). Overall, these results suggest that during inhibition days, rats were less able to choose the higher-value reward during original learning, which may have led to relatively better performance in subsequent blocks as discussed later.

### Forced-choice behavior was not significantly impacted during sessions that were fully completed

**Figure 4.**
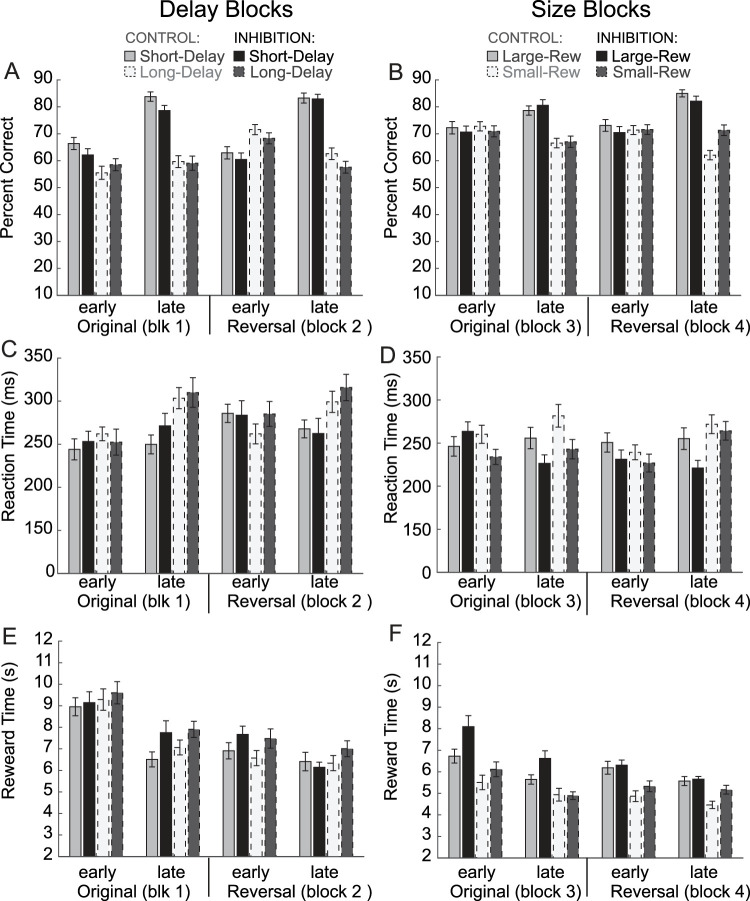
Behavioral analyses across completed sessions, with behavior during delay blocks on the left column of graphs, and size blocks on the right column. Analyses are broken down by the learning phase (early in session vs late in session once contingencies have been learned) and contingencies (originally learned contingencies vs contingency reversal). ***A***, ***B***, Average percentage of correct response on forced-choice trials during completed sessions. ***C***, ***D***, Average RTs during completed sessions. ***E***, ***F***, Average time spent consuming reward during completed sessions.

During forced-choice trials, rats had to respond in the cued direction in order to receive reward (i.e., correct trial); if rats responded in the incorrect well, no reward was delivered (i.e., error trial). As a reminder, when analyzing across all sessions, we found that inhibition disrupted forced-choice behavior (Fig. 2*E*). However, in order to be able to analyze our data by learning phase and reward value manipulations, we then conducted analyses across only completed sessions (inhibition, 36; control, 58). We found that—when solely analyzing sessions that rats were able to complete—there were no significant differences in performance on forced-choice trials. As previously reported, we found that rats tended to be more and less accurate on high- and low-value forced-choice trials, respectively (main effect of value, *F*_(1,1472)_ = 77.1; *p* < 0.0001; Fig. 4*A*,*B*; solid vs dashed). This presumably reflects their perseverance toward the fluid well associated with the more favorable reward, further demonstrating their awareness of block contingencies. As with free-choice behavior, these contingency differences are learned within blocks, and the value preferences of the rat are thus established by the end of the blocks (interaction effect between phase and value, *F*_(1,1472)_ = 81.79; *p* < 0.0001). Also noteworthy is that rats performed better in the context of size blocks—presumably because they are more motivated by obtaining larger rewards and having no delays (main effect of block type, *F*_(1,1472)_ = 30.89; *p* < 0.0001). Thus—similar to our findings in previous studies—we demonstrate that by the end of blocks, rats performed high-value forced-choice trials with higher accuracy and perform better during size blocks. Remarkably, there was no significant main effect of inhibition (*F*_(1,1472)_ = 0.81; *p* = 0.37) nor interactions between manipulation and value (*F*_(1,1472)_ = 1.38; *p* = 0.24), phase (*F*_(1,1472)_ = 0.40; *p* = 0.53), or block type (*F*_(1,1472)_ = 2.91; *p* = 0.09). Thus, when considering only completed sessions, accuracy on forced-choice trials was not impacted by inhibition. This suggests that the observed effects on accuracy across all sessions (Fig. 2*E*) were driven by session completion—a finding which is also consistent with Figure 2*H*, wherein higher heightened attention is conducive to higher accuracy.

### ACC inhibition did not reduce the motivational modulation of RT

Similar to accuracy on forced-choice trials, RTs (odor offset to odor port exit) can reflect motivational biases toward more favorable reward (Fig. 4*C*,D). That is, as rats develop behavioral biases toward fluid wells associated with higher-valued reward, RTs become faster and slower for responses into high- and low-value wells, respectively [main effect of phase (*F*_(1,1472)_ = 4.91; *p* = 0.0269) and value (*F*_(1,1472)_ = 6.05; *p* = 0.014), and these factors interact (*F*_(1,1472) _= 10.22; *p* = 0.0014)]. Furthermore, rats become faster overall during size blocks compared with that during delay blocks. Interestingly, rats were even faster on “small” reward trials, which are identical to “short”-delay trials (i.e., one sucrose bolus, no delay), suggesting that size blocks are generally more motivating (main effect of block type, *F*_(1,1472)_ = 19.52; *p* < 0.0001). Notably, the task design is constructed in this way to promote the completion of all four trial blocks.

As above for the percentage of correct response, we analyzed the completed sessions only and found that inactivation of the ACC did not globally impact RTs—as evidenced by no main effect of inhibition (*F*_(1,1472)_ = 0.84; *p* = 0.3591)—nor did it impact the influence that reward value had on RTs (no interaction between manipulation and value; *F*_(1,1472)_ > 0.0001; *p* = 0.9509). The only significant factor that included manipulation (control vs inhibition) was the interaction between inhibition and block type (*F*_(1,1472)_ = 4.54; *p* = 0.0332), which can be explained by overall faster RTs during size compared with delay blocks. With that said, post hoc comparisons conducted for individual trial types produced no significant effects (*t* tests comparing control vs inhibition for early and late phases and short, long, large, and small trial types; *t*_(92)_'s > 1.46; *p*'s > 0.1466) suggesting that the interaction emerged from both a slowing and acceleration of RTs in delay and size blocks, respectively.

### ACC inhibition did not reduce the time spent consuming reward

Finally, it might be argued that ACC inactivation decreases the desirability or motivational value associated with reward. If true, then rats might spend less time consuming reward after delivery, especially late in sessions. To address this issue, we plotted the time spent in the well after reward delivery (i.e., reward delivery onset to well exit) for all four blocks in the order that they were performed (Fig. 4*E*,F). As expected, the time spent in the well after the onset of reward delivery was longer for large compared with that for small reward (Fig. 4*F*, solid vs dashed). It is no surprise that it took rats longer to drink two drops of sucrose compared with one. However, and more interestingly, rats spent the most time in the well following reward delivery early in the first block of trials—when they first initiated the task and thus were most thirsty (Fig. 4*E*; “original”); this declined over the course of the four trial blocks [main effect of phase (*F*_(1,1472)_ = 33.47; *p* < 0.0001) and block type (*F*_(1,1472)_ = 98.91.54; *p* < 0.0001)]. Importantly, decreases in the time spent in the well across the four trial blocks were not more prominent during ACC inhibition, as evidenced by no significant interactions between inhibition and phase (*F*_(1,1472)_ = 0.03; *p* = 0.8569) or block type (*F*_(1,1472) _= 0.02; *p* = 0.8936). Instead, we found a main effect of inhibition (*F*_(1,1472)_ = 9.64; *p* = 0.0019), demonstrating that rats tended to spend more time in the well postreward delivery when the ACC was inhibited. We speculate that this result reflects the rat's drive to consume as much reward as possible given their difficulties in initiating and completing trials; regardless, at the very least, it shows that ACC inhibition did not reduce the desirability of the reward.

### DDM

The DDM posits that an organism makes decisions by dynamically gathering evidence from its environment (Ratcliff and McKoon 2008; Ratcliff et al., 2016). Evidence about a stimulus accumulates in a noisy fashion at a set of average rate (drift rate) from a potentially biased starting point until reaching a threshold (decision boundary) for one of the options. Drift rate, starting point, and the distance between decision boundaries are the model parameters.

There are multiple underlying computations that could explain choice effects. For example, impaired accuracy could be explained by a slower evidence accumulation process—which would be reflected by a smaller drift rate—or by less evidence needing to be accumulated before a decision is made. Likewise, faster RTs and higher accuracy observed in size blocks might be driven by larger drift rates or a stronger bias toward the more valued option. Using the DDM allows us to disentangle between those competing hypotheses and provide insights into the underlying computational processes that are giving rise to these observed behaviors. In addition, the model allows for inferences to be made regarding specific dimensions of decision-making process that might be conflated or not be intuitive when examining raw behavior (White et al., 2010; Lerche and Voss, 2020).

**Figure 5.**
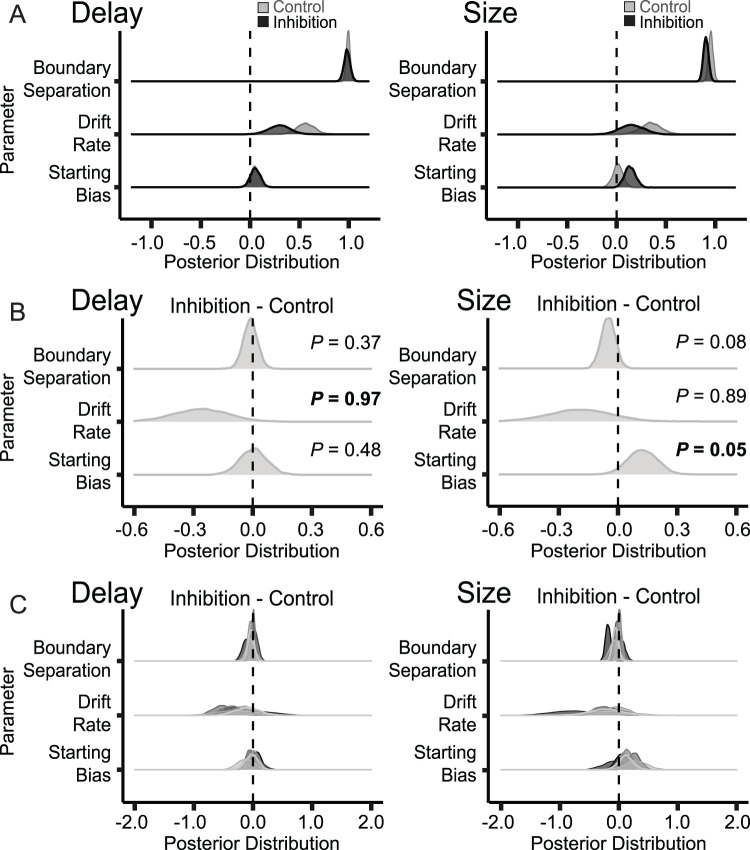
DDM parameter distribution and manipulation (e.g., inhibition vs control) difference for delay (left column) and size (right column) trials. ***A***, Inhibition (black) and control (gray) posterior distributions for each parameter. ***B***, Contrasts between inhibition and control session averages and probabilities that the contrasts are different from 0 in the hypothesized direction (>0, testing for the probability that the parameter is greater for inhibition than control, or <0, testing for the probability that the parameter is lower for inhibition than control). Probability values greater than 0.95 indicate a significant effect in the hypothesized direction; probability values lower than 0.05 indicate a significant effect in the opposite direction than hypothesized; probability values closer to 0.50 indicate no differences between the conditions. ***C***, Contrasts between inhibition and control sessions for individual rats (each rat is represented by a different shade of gray).

The marginal posterior distributions of boundary separation, drift rate, and bias are depicted in Fig. 5. Figures display the parameter distribution (Fig. 5*A*) and the average difference between inhibited and control sessions along these parameters (Fig. 5*B*) on delay (left column) and size (right column) trials, where *P* indicates the probability that the inhibition distributions are shifted relative to controls—specifically testing for larger boundary separation (positive shift), reduced drift rate (negative shift), and reduced bias toward the better option (negative shift) during inhibited relative to control sessions. As shown, the drift rate was shifted in both delay and size blocks but only significantly on delay blocks (the probability of negative shift was 0.98). These decreased drift rates suggest that on inhibition days, rats accumulated information more slowly than on control days (i.e., lower drift rates).

Finally, we examined boundary separation and starting bias. We found no differences in boundary separation; however, during size blocks, there was a higher start bias during ACC inhibition (the probability of a negative shift was 0.04, meaning that a positive shift, increase in start bias, occurred with a probability of 0.96).

## Discussion

We have previously published two studies highlighting the role of the ACC in shifting the allocation of attentional resources toward behaviorally relevant stimuli following outcome expectancy violations and how disruption of these attentional signals via cocaine self-administration leads to impairments in decision-making and cognitive flexibility (Bryden et al., 2011; Vázquez et al., 2020). In these studies, we showed that ACC firing was correlated with behavioral shifts in attention following unsigned prediction errors—as rats updated previously learned behavior–outcome contingencies. Additionally, we found that chronic cocaine self-administration attenuates attention-related ACC signals, which correlated with a variety of task impairments **(**Vázquez et al., 2020). From these results, we had hypothesized that ACC signaling must give rise to specialized functions related to attention and cognitive control that promote optimal reward-guided decision-making when contingencies change. Here, we tested this prediction by transiently inactivating the ACC using optogenetics. We found that ACC inactivation had a profound impact on the rats' basic abilities to initiate and complete trials, which resulted in fewer rewards and completed sessions overall. Importantly, RTs, MTs, and reward consumption averaged across trial types and choices were not impaired, suggesting that these deficits in the rats' ability to initiate, complete, and attend to the task were not due to motor control or motivational deficits.

Our main hypothesis going into this study was that the functional ACC was necessary for allocating attention to reward-predicting cues so that value contingencies can be learned, consistent with studies that have implicated the ACC in reward processing, conflict monitoring, error detection, and allocation of attention (Carter et al., 1998; Barch, 2001; Braver, 2001; Botvinick et al., 2004; Holroyd et al., 2004; Kerns et al., 2004; Weissman, 2004; Yeung et al., 2004; Aarts et al., 2008; Totah et al., 2009; Hayden et al., 2011; Hyman et al., 2013; Laubach et al., 2015; Newman et al., 2015; Wu et al., 2017; Soltani and Izquierdo, 2019). In our study, we found that ACC inactivation severely impeded continuous task performance, as demonstrated by reductions in trial initiations, as well as in trial and session completions; in turn, this severely impaired the task accuracy. Furthermore, LO latency—the measure of attention to the task we have used throughout our studies—was significantly slower on inhibition days; consistent with our previous research, the performance on the task was correlated with this behavioral proxy of attention to the task (Vázquez et al., 2020).

When analyzing across only completed sessions, the only deficits that we observed were on free-choice trials during the original learning, reflecting slower accumulation of evidence (i.e., smaller drift rate) and/or weaker response–outcomes associations, which might result from changes in attention (Dayan et al., 2000; Krajbich et al., 2010, 2012; Nunez et al., 2017; Retzler et al., 2020). Arguably, weaker associations formed in the context of original learning might have given rise to better choice performance in size blocks (i.e., easier to override previously learned associations). That is, in the DDM, impaired learning seems to arise from a general reduction in drift rates (albeit only significantly so for delay blocks), while the easier tendency to override previously learned associations during size blocks—which occurred later in sessions—may be explained by the heightened bias during these blocks. Regardless of the specific interpretation, during inhibition, drift rates and bias contributed less and more, respectively, to free-choice performance. This could also indicate a reduced ability to learn through evidence accumulation, which would instead be replaced in later blocks by an increased tendency to rely on the heuristics of a biased decision.

Overall, the ACC seems to be critically involved in promoting the initiation and completion of trials and behavioral sessions, which might reflect general deficits in attention or motivation or willingness to engage in a cognitively demanding task. Motivation and attention are sometimes difficult to disambiguate. Deficits in either could explain the observed decreased rates of trial initiation and completion. However, neither explain why there were inhibition sessions which rats were able to complete and how during these completed sessions they were still able to reverse contingencies, respond appropriately on forced-choice trials, and show stronger motivation on high-value compared with that on low-value reward trials (i.e., rats were better and faster on high-value forced-choice trials). Furthermore, if anything, during completed inhibition sessions, rats were faster during size blocks relative to delay blocks and actually spent more time in the fluid well consuming reward compared with that of during control sessions. Thus, there were sessions during which rats were able to attend to (i.e., respond to cues, reverse contingencies) and process motivational cues (i.e., better/faster for high value) during ACC inhibition. Although it is difficult to determine whether the observed deficits reflect general decreases in attention, it is clear that rats seem to be less engaged with the task during inhibition sessions.

Inhibition of the ACC resulted in deficits that were present throughout the entire session during both LED+ and LED− trials (i.e., did not differentially impact behavior on a trial-by-trial basis but rather had a global impact on task engagement). Thus, it appears that inhibiting ACC on only 50% of trials is sufficient to place rats in a generally less engaged state. Studies using halorhodopsin have shown that it can enable prolonged hyperpolarization and suppress action potentials on a timescale of minutes, with slow recovery from inactivation (Mahn et al., 2018; Zhang et al., 2019), which could also explain why behavioral effects were present during both LED-on and LED-off trials.

The lack of engagement throughout the task might reflect the inability to allocate cognitive resources to complete trials but might also specifically reflect the inability to exert effort to complete a task that is cognitively demanding. It has been shown that rats with ACC lesions are less able to exert effort to obtain large reward (Walton et al., 2003; Holec et al., 2014; Hosking et al., 2014). For example, studies have found that rats with ACC lesions exhibit a decreased inclination to scale a physical barrier in order to acquire a large reward, opting instead for a small reward that requires no effort to acquire (Walton et al., 2003; Holec et al., 2014); furthermore, in separate case studies involving human subjects with bilateral ACC lesions, researchers found that these individuals exhibited motivational and attentional impairments, as well as akinetic mutism (Barris and Schuman, 1953; Laplane et al., 1981). Although our task does not require physical exertion, it is a cognitively demanding task in that rats must follow basic trial structure (i.e., respond when houselights are on, maintain hold, respond to one of the wells) and learn, track, and update response–outcome associations—which vary across two reward dimensions throughout four 60-trial blocks—all while following forced-choice rules. Perhaps the ACC is particularly important when rats need to sustain the attention in a task that requires considerable cognitive effort or complexity.

In summary, we found that ACC inhibition reduced trial initiation and both session and trial completion and deleteriously impacted the task performance without impacting RTs or MTs. Finally, choice selection during inhibition sessions was biased toward the direction associated with the originally learned response–outcome association. Overall, behavior on inhibition days was suboptimal, in that fewer trials were completed and better rewards were selected less often. While these results may reflect the ACC's role in attention, we speculate that it reflects that the ACC is necessary to initiate and stay on task, especially during cognitively demanding tasks. Importantly and consistently, one of the hallmarks of attention-deficit/hyperactivity disorder—which has been associated with ACC hypofunction (Rubia et al., 1999; Ernst et al., 2003; Bush et al., 2005; Konrad et al., 2006; Pliszka et al., 2006; Zilverstand et al., 2017) and structural abnormalities (Seidman et al., 2006; Makris et al., 2008, 2010; Qiu et al., 2011; Vogt, 2019; Yu et al., 2023)—is difficulty with task initiation (Lauth et al., 2006; Mills et al., 2018; Dieckhaus et al., 2021).
